# Relationship of cognitive function in patients with schizophrenia in remission to disability: a cross-sectional study in an Indian sample

**DOI:** 10.1186/1744-859X-6-19

**Published:** 2007-07-30

**Authors:** Rajeev Krishnadas, Brian P Moore, Ajita Nayak, Ramesh R Patel

**Affiliations:** 1 Tranwell Unit, Queen Elizabeth Hospital, Gateshead, Tyne and Wear, UK; 2 BYL Nair Hospital, AL Nair Road, Mumbai, India; 3 Bhatia Hospital, Tardeo, Mumbai, India

## Abstract

**Background:**

Cognitive deficits in various domains have been consistently replicated in patients with schizophrenia. Most studies looking at the relationship between cognitive dysfunction and functional disability are from developed countries. Studies from developing countries are few. The purpose of the present study was to compare the neurocognitive function in patients with schizophrenia who were in remission with that of normal controls and to determine if there is a relationship between measures of cognition and functional disability.

**Methods:**

This study was conducted in the Psychiatric Unit of a General Hospital in Mumbai, India. Cognitive function in 25 patients with schizophrenia in remission was compared to 25 normal controls. Remission was confirmed using the brief psychiatric rating scale (BPRS) and scale for the assessment of negative symptoms (SANS). Subjects were administered a battery of cognitive tests covering aspects of memory, executive function and attention. The results obtained were compared between the groups. Correlation analysis was used to look for relationship between illness factors, cognitive function and disability measured using the Indian disability evaluation and assessment scale.

**Results:**

Patients with schizophrenia showed significant deficits on tests of attention, concentration, verbal and visual memory and tests of frontal lobe/executive function. They fared worse on almost all the tests administered compared to normal controls. No relationship was found between age, duration of illness, number of years of education and cognitive function. In addition, we did not find a statistically significant relationship between cognitive function and scores on the disability scale.

**Conclusion:**

The data suggests that persistent cognitive deficits are seen in patients with schizophrenia under remission. The cognitive deficits were not associated with symptomatology and functional disability. It is possible that various factors such as employment and family support reduce disability due to schizophrenia in developing countries like India. Further studies from developing countries are required to explore the relationship between cognitive deficits, functional outcome and the role of socio-cultural variables as protective factors.

## Background

Neurocognitive dysfunction has been postulated as a core deficit in many major mental illnesses [1-3]. Rather than a gene that is linked to a specific illness, it is proposed that deficits in information processing could be endophenotypes that are inherited [4]. A constellation of these core cognitive deficits (endophenotypes) in various combinations and severity have a role in the emergence of well established psychopathology of the disease. It has been suggested that people with a lower cognitive reserve tend to have psychosis-like experiences more readily compared to those with a greater cognitive reserve [5].

Cognitive deficits in various domains have been consistently replicated in patients with schizophrenia [1]. Longitudinal studies have confirmed that most deficits are trait abnormalities, persistent and stable over time [6]. In patients with schizophrenia, delusions and hallucinations could arise as a result of deficits in cognitive functions involving perceptual and attributional biases [7,8].

People with schizophrenia are known to have problems with initiation and maintenance of social activity. Access to public funds early in the illness and dependence on them is an indicator of the severity of functional deterioration. This deterioration takes place early in the illness process [9]. Data from industrialized nations show that only around 10% of patients diagnosed with schizophrenia are working in full-time employment.

Most studies that link cognitive deficits to functional outcome in schizophrenia support the notion that neurocognitive function predicts social and occupational function. Measures of immediate memory, delayed memory, and executive function have been found to predict functional outcome with small to medium effect size [10]. Moreover, cognitive function has been found to be a better predictor of functional outcome than symptom levels [11].

Cross-cultural studies have found that outcome of schizophrenia is better in developing countries [12,13]. The most recent data from the Madras longitudinal study, which followed up patients with first episode schizophrenia for 20 years, found that more than 75% of patients remained employed at the end of 20 years. The reasons postulated for this difference in outcome being the possibility of a biological difference, or the presence of socio-cultural factors that protect from functional deterioration [14].

Although cognitive deficits in patients with schizophrenia have been shown in the Indian population [15-17], very few have looked at the relationship between cognitive dysfunction and functional disability. The present report documents a cross-sectional study that aimed to compare the cognitive function in people suffering from schizophrenia, but were in remission, with that of normal subjects. We explored a relationship between the demographic and illness variables, cognitive function and functional outcome as measured by the Indian disability evaluation and assessment scale (IDEAS) [18].

## Methods

The study took place in the Department of Psychiatry, BYL Nair Hospital, a municipal general hospital in Mumbai, India. It received ethical approval from the institutional review board at Nair Hospital. Written informed consent was obtained from all subjects to participate in the study, and they were free to withdraw at any time.

### Subjects

A total of 25 subjects were recruited into the study from the outpatient clinics of BYL Nair hospital, Mumbai. We included patients aged 18–60 years old, with a diagnosis of schizophrenia according to DSM IV [19] criteria, made by an investigator (RK) and confirmed by senior psychiatrists (AN and RRP). The diagnosis of schizophrenia was made at clinical interview and augmented by discussion with relatives and a review of clinical notes. Similarly, co-morbid Axis I and II diagnoses were excluded. This was confirmed from the case notes and history from the relatives. Remission was ascertained clinically using a cut off total score of 8 with a score of 2 or less on individual items on the brief psychiatric rating scale (BPRS) [20] and scale for the assessment of negative symptoms (SANS) [21]. Subjects with significant physical and neurological illness; a history of stroke or head trauma in the past; a history of alcohol or drug misuse; a mini mental state examination (MMSE) score of less than 28; a history of receiving electro-convulsive therapy (ECT) in the past six months; evidence of co-morbid Axis I or II psychiatric disorder; and a childhood history suggestive of mental retardation (DSM IV) [19] were excluded from the study. Demographic details, illness variables and treatments were recorded using a semi-structured proforma. Controls were healthy subjects who had no family history of major psychiatric illness in a first-degree relative. They conformed to the same exclusion criteria as the participants with schizophrenia.

### Procedure

All subjects who were included in the study were assessed once using a battery of tests for cognition that lasted from 1 h 15 min to 2 h. Assessments were performed in a fixed order in a quiet room by RK. At the subject's request, a short break was permitted halfway through the assessment. Patients were not allowed to smoke or consume stimulant drinks during the assessment. The last dose of medication was taken at least 6 h before the testing.

### Cognitive function

#### The PGI memory scale

The PGI memory scale [22] is part of the PGI battery of brain dysfunction, developed at PGIMER, Chandigarh, India. The battery is administered in Hindi, the first language of most subjects, and has been developed and validated for use in the Hindi-speaking population. It includes 10 subtests, of which we used the 7 that measured various aspects of verbal and visual memory. These included forward and backward digit spans, 1 min delayed recall of a word list, immediate recall of sentences, retention of similar word pairs, retention of dissimilar pairs, and visual retention and visual recognition. We excluded three tests from the analysis (recent memory, remote memory and mental balance test) because they demonstrated a ceiling effect.

#### Trail making tests A and B

Trail making tests A and B have been commonly used and validated in the Indian population [23]. Trail shift scores were calculated by subtracting the time taken on Trail making A from the time taken on Trail making B test.

#### Rey-Osterrieth complex figure test

The Rey-Osterrieth complex figure test [24] is used to evaluate both visuo-constructional ability and visual memory. The accuracy of the immediate and the 30 min recall versions were assessed using the standardized scoring system.

#### Frontal Assessment Battery (FAB)

The frontal assessment battery (FAB) [25] is a short cognitive and behavioral battery that assesses frontal lobe function. It has six subtests, each of which has been shown to correlate with frontal lobe metabolic activity measured by 18 flurodeoxyglucose on a PET scan [26]. The subtests are similarities (tests conceptualization and abstract reasoning), verbal fluency (tests word generation), Luria's motor series test (tests motor series programming requiring temporal organization, maintenance and execution of successive action), conflicting instructions (tests sensitivity to interference, where verbal commands conflict with visual sensory information), go/no go (tests the ability to inhibit an inappropriate response to tasks anticipated to elicit a false alarm motor response), and prehension behavior (checks the environmental autonomy).

### Disability assessment

The Indian Disability Evaluation Assessment Scale (IDEAS) was developed by the rehabilitation committee of the Indian Psychiatric society [18]. It assesses the individual under four domains: self care, interpersonal activities (social relationships), communication and understanding, and occupation, including performance at employment/housework/education. Each item is scored between 0–4, i.e., from no to profound disability. Adding the scores on the four items gives the 'total disability score'. IDEAS has been field tested in nine centers all over India. Internal consistency between items was good, with a Cronbach's alpha value of 0.87. It has good face and criterion validity, established by comparing IDEAS with the Schedule for the Assessment of Psychiatric Disability (SAPD), which has been standardized in the Indian population.

### Statistical analysis

Data was analyzed using SPSS v 12 [27]. Data were checked for normality using the Kolmogorov-Smirnov test. When it was found to be non-normal, data was analyzed using non-parametric methods. Difference between groups was tested using unpaired t tests or the Mann-Whitney U test where appropriate. Categorical data was compared using Chi-square tests, and where the expected value was less than 5 in a cell, an exact test on unordered contingency tables was used [28]. Associations between socio-demographic data, illness variables, disability score and neurocognitive performance were explored using the Spearman's correlation, as most data on cognitive tests did not assume normality. To reduce the risks of type 1 error from repeated hypothesis testing, a significance level of p ≤ 0.01 was adopted on all tests.

## Results

### Subject characteristics

There were a greater proportion of females in the schizophrenia group compared to the control group, but the difference was not statistically significant. The mean age of the patient group was higher than the controls but the number of years of education was slightly higher in the control group. The mean duration of illness was 11.3 ± 5.8 years, ranging from 2 to 20 years. A total of 24% of the patients were unemployed, whereas there were no unemployed subjects in the control group, a difference that was statistically significant (Chi-square = 3.6E-07, p < 0.001). Mean BPRS, Hamilton rating scale for depression (HRSD) and the SANS scores are shown in Table 1. Although the mean scores were clinically low, they were found to be statistically higher than the control population. Mean score on total IDEAS was 2.16 (SD ± 2.267).

**Table 1:** Demographic and illness characteristics of groups

	GROUP			
			
	Schizophrenia	Control		p
Gender:				
Female, n (%)	9 (36)	7 (28)	0.368	0.544
Male, n (%)	16 (64)	18 (72)		
Age, years: mean (sd)	40.16 (8.153)	35.48 (5.49)	t = 2.380	0.021
Years in education	9.08(1.470)	9.92 (1.038)	t = -2.335	0.024
Duration of illness, mean (SD)	11.32(5.8)	-		
Employment:				
Unemployed	6	0	3.6E-07*	<0.001
Self employed	6	0		
Govt/private salaried	6	22		
Home maker	7	3		
BPRS, mean (SD)	3.68 (1.249)	0	t = 14.732	<0.001
HRSD, mean (SD)	1.36 (.952)	0	t = 7.141	<0.001
MMSE, mean (SD)	29.80 (.408)	29.92 (.277)	t = -1.216	0.230
SANS, mean (SD)	2.68 (.690)	0	t = 19.4	<0.001
IDEAS, mean (SD)	2.16 (2.267)	-		
Medication:				
Typical antipsychotics	22(88)	-		
Atypical antipsychotics, n (%)	1 (4)	-		
Combination of antipsychotics	2 (8)	-		
Benzodiazepine, n (%)	8 (32)	-		
Anticholinergics, n (%)	24 (96)	-		
Antidepressant, n (%)	3 (12)	-		

*Exact test on unordered contingency tables.

Details of medication are shown in Table 1. A total of 96% of the patient group was on a typical antipsychotic, and an equal number were on an anticholinergic medication. 32% of the patients took a benzodiazepine.

### Neurocognitive function

Results are reported according to the domains of cognition tested (Table 2). Patients with schizophrenia scored less well than controls on all tests of cognition, except visual recognition.

**Table 2:** Comparison of cognitive tests between the two groups

	CONTROLS SCHIZOPHRENIA			
			
TESTS	Mean (SD)	Mean (SD)	Z (U)*	p
Digit forward	5.92 (0.28)	3.52 (0.65)	-6.442 (2.000)	<0.001
Digit backward	4.36 (0.49)	2.32 (0.69)	-6.129 (8.000)	<0.001
Immediate recall	11.88 (0.44)	3.84 (0.99)	-6.431 (0.000)	<0.001
Delayed recall	9.92 (0.28)	6.52 (1.94)	-6.262 (8.000)	<0.001
Verbal retention: similar pairs	5.00 (0.00)	3.44 (0.77)	-5.981 (37.500)	<0.001
Verbal retention: dissimilar pairs	14.84 (0.37)	5.96 (1.31)	-6.334 (0.000)	<0.001
Visual retention	12.88 (0.33)	6.24 (2.18)	-6.353 (0.000)	<0.001
Visual recognition	9.96 (0.20)	9.68 (0.48)	-2.551 (225.00)	0.011
Trail making A	39.80 (3.00)	77.24 (18.87)	-5.973 (6.000)	<0.001
Trail making B	79.72 (7.71)	150.76 (17.32)	-6.074 (0.000)	<0.001
Trail shift	39.92 (6.33)	73.52 (27.43)	-4.980 (56.000)	<0.001
Total FAB	9.60 (1.35)	18.00 (0.00)	-6.507 (0.000)	<0.001
ROCFT 1	35.36 (0.81)	31.64 (1.32)	-6.121 (2.500)	<0.001
ROCFT 2	27.72 (1.57)	14.36 (2.51)	-6.085 (0.000)	<0.001

*Mann-Whitney U test. Mean (SD) reported for ease of interpretation of data. All tests considered significant if p < 0.01.

### Attention and vigilance

Trail making test A has been found to be a good measure of attention and vigilance (sustained attention). People with schizophrenia took significantly longer time than controls to complete the task.

### Immediate and working memory

Patients with schizophrenia scored significantly lower on immediate sentence recall and digit span tests. Performance on similar and dissimilar pair retention was worse in the patient group. Patients fared worse on the visual retention subtest of the Postgraduate Institute memory scale (PGIMS) and the Rey-Osterrieth complex figure test (ROCFT) immediate visual recall.

### Delayed memory

Delayed recall assesses verbal episodic memory. Patients with schizophrenia recalled significantly less number of words compared to normal controls. Visual episodic memory on ROCFT recall at 30 min was worse in patients with schizophrenia, compared to controls. There was no difference in scores of visual recognition test between the patients and controls.

### Executive funtion

Patients with schizophrenia fared worse than controls on the frontal assessment battery. The only test in the FAB, on which the two groups scored similarly, was the environmental autonomy subtest. Reverse digit span and Trail making B test also tests the executive function, i.e. the ability to manipulate data 'online'. On Trail shift, the difference between the performance on Trail A and B (Trail B-A), was significantly worse in patients with schizophrenia. These findings suggest a frontal executive dysfunction. Correlation of demographic details, illness variables and disability with cognitive function.

To compensate for multiple testing, we considered an association to be significant only if the correlation coefficient was significant at p ≤ 0.01. No significant association was found between age, duration of illness, number of years in education and scores on the neurocognitive tests (Table 3). There was no significant meaningful correlation between the scores on the tests and the scores on SANS, BPRS and HRSD. There was also no significant correlation between negative and positive symptoms score and score on IDEAS. Although there was no significant relationship between scores on the cognitive tests and scores on IDEAS, the negative correlation between the scores on backward digit span and immediate recall and the score on IDEAS showed a trend towards significance at p = 0.017 and 0.014 respectively.

**Table 3:** Correlation (Spearmans) among variables

	Age	No. of years in education	Duration of illness in years	Total IDEAS	HRSD	BPRS	SANS
Digit forward	0.242	0.011	0.119	-0.396*	0.298	-0.131	0.075
Digit backward	0.321	0.109	0.337	-0.474*	0.043	0.065	0.102
Immediate recall	0.035	-0.086	-0.132	-0.483*	0.395	-0.127	0.226
Delayed recall	0.350	-0.063	0.307	-0.403	0.149	-0.065	-0.175
Verbal retention: similar pairs	0.259	-0.002	0.078	-0.385	0.399*	-0.286	0.145
Verbal retention: dissimilar pairs	0.548**	-0.136	0.317	-0.274	0.306	-0.139	0.209
Visual retention	0.139	0.326	0.089	-0.291	0.081	0.042	-0.284
Recognition	-0.197	-0.045	-0.137	-0.301	0.243	0.012	-0.078
Trailmaking A	-0.392	-0.003	-0.280	-0.027	0.316	-0.092	0.206
Trailmaking B	-0.141	0.054	-0.158	-0.077	-0.073	0.091	-0.585**
ROCFT 1	-136	0.040	-0.244	0.198	0.085	0.240	0.082
ROCFT 2	-0.140	0.087	-0.167	0.160	0.053	-0.116	0.291
Total FAB	-0.106	0.027	-0.178	-0.136	0.193	-0.260	0.096

*p < 0.05; **p < 0.01. Correlations considered significant if p < 0.01.

## Discussion

### Cognitive dysfunction

Our study replicates the findings of numerous studies that demonstrated the presence of neurocognitive deficits in patients with schizophrenia on most domains tested, including attention, vigilance, immediate memory, working memory, delayed memory and executive function [1].

A recent study from India compared the cognitive functioning of 100 symptomatic subjects with chronic schizophrenia to equal number of normal controls. It found that people with schizophrenia performed worse on all cognitive tests involving memory, attention and executive function [15]. The findings of the present study show that cognitive deficits are persistent even during periods of remission, suggesting the 'trait' nature of the deficits. The similarity of cognitive deficits across cultures also indicates that the deficits could constitute 'traits' of the illness.

### Memory deficits in Schizophrenia

Poor performance by the patients on digit span and immediate recall of sentences suggests a deficit in working memory model as proposed by Baddeley [29]. Semantically paired items on a word list did not improve performance on immediate recall. This is in keeping with earlier findings, which suggested that phonological similarity is more important than semantic similarity in short-term memory. In contrast, long-term memory depends more strongly on meaning and phonological similarity is less important. Deficits on immediate visual recall of the ROCFT and visual retention suggest a deficit in the 'visuospatial sketchpad' on Baddeley's model. A deficit in the 'central executive' is suggested by the poor performance of the patients on the Trail making B test. Patients performed less well on Trail making A test, which could indicate that they had attention deficits. Although reduced attention could explain many cognitive deficits in the patient groups, their poorer performance on 'trail shift' indicates that their executive deficits might be partially independent of the attention deficits. Poor performance on 1 min verbal delayed recall and 30 min recall on the visual ROCFT suggests a deficit in circuits involving delayed memory.

### Demographic factors, Illness variables and cognition

The present study found no relationship between age, duration of illness, psychopathology and cognitive function, suggesting that the deficits are trait deficits and are stable over the course of the illness. Previous longitudinal studies have confirmed the stable nature of cognitive deficits in patients with schizophrenia [6]. Srinivasan et al. reported a relationship between age, duration of illness, duration in formal education and neurocognitive function in a sample of Indian patients [15]. There is some evidence to suggest that older patients with schizophrenia (>65) show a progressive decline in cognitive functioning compared to younger patients [30].

### Symptomatology and functional outcome

Previous studies performed in the Western population have found an association between negative symptoms and a poor functional outcome. Brier et al. found that negative symptoms measured using SANS correlated to poor functional outcome measured using the Global assessment scale [31]. Johnstone et al. found that social withdrawal predicted functional outcome [32]. One small study, which included 19 patients with schizophrenia, found that symptoms measured using BPRS and SANS did not predict outcome [33].

A recent study that measured psychopathology using the Positive and Negative Syndrome Scale (PANSS) and disability using IDEAS in an Indian population showed a positive correlation between scores on all three PANSS subscales and scores on IDEAS, suggesting a relationship between levels of symptomatology and disability. The study did not measure cognitive functioning of the patients. [34]. Our study did not show a relationship between symptomatology and functional outcome. This could be explained by the low level of symptoms in the study population, because we recruited only patients who fulfilled the criteria for remission.

### Cognitive function and disability

The present study did not find a strong association between cognitive function and disability. Although this is in contrast to most previous studies, a few studies have shown similar results. Johnstone et al. in a study of 137 first episode patients found that neurocognitive function measured using the Peabody picture vocabulary test and digit symbol substitution test did not correlate with occupational functioning [32]. Addington et al. found no association between cognitive measures and social functioning in 30 patients with schizophrenia [35]. The reason for these differences is unclear.

Our measured correlates in many cases were high, often up to 0.45, which would explain 20% of the total sample variance. However, we corrected for multiple comparisons to decrease the chance of false positive. But in doing so, discounted correlates with p value lying between 0.05 and 0.01. Hence, few correlates were regarded as significant.

These findings could also be attributed to 75% of the sample that remained employed in spite of the significant cognitive deficits. Similar employment rates have been shown in previous studies from India [14]. The findings in the present study emphasize the importance of protective factors that could play a role in the functional outcome of patients in spite of cognitive deficits. Although there is no strong evidence, socio-cultural factors have been implicated as a reason for better prognosis in developing countries. Families in India, play a major role in patient management. They take up the responsibility for the patient's compliance with medication and support them in times of crisis and need, functions, which in the West are carried out by assertive outreach teams, crisis teams and community mental health teams, and are believed to improve outcome [36]. Further, the absence of access to social security and public funds in the form of disability allowance might have contributed to the pressure to earn for a living and hence the high employment rate. Employment might offer the patient a form of practical social and functional rehabilitation. The possibility of finding a job in the unorganized sector as street vendors and manual labourers make it easy for people to earn a living and remain functional from an employment point of view [36]. All the patients who participated in our study were living with their families and had good support. Further studies are required to confirm the hypothesis of socio-cultural factors impacting on the outcome of schizophrenia [37].

The study has a number of limitations. First of all, the small sample size might have led to a type 2 error. We did not test the premorbid intelligence (and hence the cognitive reserve) of the patients, although any history suggestive of a developmental delay was excluded from the participants. All patients in our study were on medications, including typical antipsychotics, anticholinergics and benzodiazepines, known to cause cognitive deficits. Although previous studies on first episode drug naïve patients have confirmed that the deficits are independent of medication, the possibility of the medications worsening the cognitive functioning cannot be ruled out [38]. Recent consensus suggests a time criterion of 6 months in defining remission [39]. The present study did not confirm remission prospectively. The investigator who administered the test was not blind to the diagnosis, and this might have lead to unintentional observer bias. It is not known how generalisable the data is to community, where symptoms might not be as controlled as in the population tested.

## Conclusion

Persistent cognitive deficits are seen in patients with schizophrenia under remission when compared to normal controls. The cognitive deficits are not associated with symptomatology and disability. It is possible that various factors such as employment and family support reduce disability due to schizophrenia in developing countries like India. These factors either compensate for the deficits or act as natural social and functional remediation. Further longitudinal studies from developing countries are required to explore the relationship between cognitive deficits, functional outcome and the role of socio-cultural variables as protective factors.

## Competing interests

The author(s) declare that they have no competing interests.

## Authors' contributions

RK was involved in the conceptualization of the study, literature review, recruiting patients, data collection, statistical analysis and interpretation of results. PBM was involved in review of the manuscript including extensive revision of interpretation and discussion of the results. AN and RRP reviewed the methodology, contributed to recruiting patients, confirming the diagnosis and interpretation of results.
